# Editorial: Case reports in cardiovascular genetics and systems medicine: 2022

**DOI:** 10.3389/fcvm.2023.1282147

**Published:** 2023-09-11

**Authors:** Neil V. Morgan

**Affiliations:** Institute of Cardiovascular Sciences, College of Medical and Dental Sciences, University of Birmingham, Birmingham, United Kingdom

**Keywords:** case-report, clinical cases, genetics, cardiovascular disease, rare variants, genotype-phenotype

**Editorial on the Research Topic** Case reports in cardiovascular genetics and systems medicine: 2022

Case reports remain a very useful resource especially for rare genetic conditions where only a handful of reported mutations maybe reported. Indeed, it has previously been reported that case-reports, are a compendium of useful ideas for our daily activity in the context of clinical management of patients (1).

But more specifically for rarer genetic diseases, there may be situations where an additional family with a distinct mutation is needed to prove the disease gene(s) is linked to the phenotype of the specific genetic disorder. Further, the clinical work up of rare cases will allow for genotype-phenotype correlations. In situations where rare genetic variants are classed as Variants of Uncertain Significance (VUS) characterising variants for clinical action is extremely important. Internationally there are many efforts ongoing to address this such as the ClinGen Gene Curation Working Group (2). This grouping has developed a method to qualitatively define the “clinical validity” of a gene-disease relationship using a semiquantitative method to assess the clinical validity of gene-disease relationships. More recently this has been applied to a well-known cardiovascular disease, Dilated Cardiomyopathy (DCM) (3). More specifically in this study they have investigated evidence-based assessment of genes in DCM where the authors carried out a large-scale analysis of 51 genes previously associated with DCM, providing strong evidence for DCM associated genes that can be used to define and improve clinical practice and management of patients. Overall case reports may also highlight new clinical findings for a particular disease/syndrome, that have not been previously documented thus can extend the phenotypic spectrum. This can be true even for genetic diseases with mutations in the same gene, known as allelic heterogeneity.

In this latest exciting series of case reports, we illustrate the value of publishing such case reports in a range of cardiovascular genetic diseases including cardiomyopathies, tetralogy of fallot (TOF), and other rare syndromes including Ehlers Danlos syndrome. The series includes 2 case reports highlighting where specific mutations in a Cardiomyopathy can be linked to sudden cardiac death, a situation where defining the genetics can not only give a reason for this devasting outcome, but also inform genetics for future cases within the same family and allowing for accurate genetic counselling. One of these cases published by Jin et al. report a variant in the Myosin Binding Protein C3 encoding *MYBPC3*, which is an apparent “silent” variant which although doesn't change the amino acid, results in altered splicing of the transcript leading to reduced expression of the gene.

An interesting case published by Wang et al. report a family with TOF, which is the most common cyanotic congenital heart disease (CHD), but only a small number of familial TOF cases have been reported to date. Using whole exome sequencing (WES) the authors report a novel heterozygous missense variant in the *MYOM2* gene, thus expanding the spectrum of the gene variants that lead to TOF.

Two other case reports are focused on Ehlers Danlos syndrome, a rare connective tissue disorder characterised by spontaneous arterial, bowel or organ rupture. In one case Manhas et al. functionally characterise a missense variant in *COL3A1*, classifying it as pathogenic. A further case of Ehlers-Danlos syndrome was reported by Taurino et al. with complex arterial findings and death at young age identified a novel *COL3A1* variant which concludes early diagnosis could lead to treatment choices, improved management, and ultimately, better outcomes.

In summary, this series of case reports include rare genetic diseases that are important to publish and extend the phenotype-genotype correlations of this collection of rare cardiovascular diseases. They highlight the challenges and value in assigning pathogenicity of the specific genetic variant where functional characterisation is often required, in order to ultimately improve patient management and introduce personalised treatments where possible.
